# A Gift of Days

**Published:** 1891-08-29

**Authors:** 


### PASSING TOPICS.

### A GIFT OF DAYS.

It was a kindly and sensible act on the part of a recent meeting of subscribers to one of the minor hospitals to show their appreciation of the efforts of their Secretary and their consideration for his health and well-being by presenting him with the substantial gift of a holiday in every week of the coming year.

So far as we know the compliment is unique, but possibly it possesses recommendations obvious enough to render it potent as an example. It says much for the zeal with which the affairs of many of our charities are administered that any such action should be called for. Workers are not usually averse from taking holidays more or less frequently, as they can get them, and we fear there are not a few callings in which a suggestion such as we are now commenting upon would be fraught with real danger. But without claiming for the administrative officials of our charitable institutions anything like a monopoly of industry, we believe that, as a class, they would compare favourably with any other in love for their work and species of unconscious self-surrender which induces them to be ever devising fresh methods of adding to their labours, and by that means to the usefulness and the resources of their institutions.

If it were not that the occasion protects them, there would be something almost amusing in the jealous guard the Secretaries and others keep over the reputations and welfare of their several charities. He would be a bold man and a blind, who would seek to disparage any given institution within the hearing of its chief officials. The latter may and probably do know well enough what weak points there are in the organization, but woe be to the outside critic who seeks to discover or to discant upon them.

It is to this strong feeling of *esprit* that the efficiency of our hospitals is in a great measure due, and perhaps in a creditable concern for its affairs, there may be, here and there, a disposition upon the part of the officials to unduly exaggerate the part they fill. Let us rest assured that we are none of us indispensable. And in regard to the matter more particularly under present notice, we may say that the machinery of a well organised institution ought to be capable of working smoothly notwithstanding a temporary withdrawal of the controlling hand.

It must be a want of belief in the depth and substantiality of the efficiency they have promoted which would lead men to think differently, and over-zealous workers would doubtless accomplish still better things if only they would take more recreation. Of course this is the merest truism, but at the holiday season it is well to be reminded of a truth which is not ugly, as truths sometimes are, but altogether pleasant and winning. Within reasonable bounds, authorities can scarcely be too liberal in the matter of holidays to those whose labours are of the wear and tear sort, and beset with such anxieties and discouragements as are involved in the maintenance of impecunious institutions. In the interests chiefly of the work carried on, but also of overworked officials in general, we commend the action of the Committee in question as eminently sound and judicious. Agricola.

